# Integrated transcriptomics and machine learning reveal REN as a dual regulator of tumor stemness and NK cell evasion in Wilms tumor progression

**DOI:** 10.3389/fimmu.2025.1612987

**Published:** 2025-06-04

**Authors:** Qingfei Cao, Junyi Li, Yunfei Zou, Changwen Xu, Huihui Tang, Meixue Chen

**Affiliations:** ^1^ Department of Urology, The First Affiliated Hospital of Jinzhou Medical University, Jinzhou, Liaoning, China; ^2^ Department of Cardiology, The First Affiliated Hospital of Jinzhou Medical University, Jinzhou, Liaoning, China; ^3^ Department of Pediatric, The First Affiliated Hospital of Jinzhou Medical University, Jinzhou, Liaoning, China

**Keywords:** Wilms tumor, tumor stemness, natural killer cell evasion, renin gene, tumor microenvironment, cancer stemness prognostic index

## Abstract

**Introduction:**

Wilms tumor (WT) is the most common pediatric kidney cancer, which presents significant therapeutic challenges, particularly in high-risk cases, due to chemotherapy resistance and immunosuppressive tumor microenvironments (TMEs). Tumor stemness and immune evasion mechanisms are implicated in poor clinical outcomes, yet the molecular drivers underpinning these processes remain inadequately understood.

**Methods:**

We employed an integrative approach combining single-cell RNA sequencing (scRNA-seq), spatial transcriptomics, bulk RNA-seq, and advanced machine learning techniques to uncover molecular regulators of tumor behavior in WT. A novel Cancer Stemness Prognostic Index (CSPI) was developed using machine learning algorithms to stratify WT patients by risk and histological subtype. Additionally, molecular docking simulations and in vitro functional assays were performed to validate the role of key regulators in tumor stemness and immune evasion, as well as to explore potential therapeutic strategies targeting these molecular drivers.

**Results:**

Renin gene (REN) emerged as a central regulator of tumor stemness and immune evasion in WT. High-CSPI tumors exhibited enhanced tumor stemness phenotypes, metabolic reprogramming (ROS/oxidative phosphorylation), and suppressed immune activity. Spatial transcriptomics revealed distinct histological subtype-specific localization of stemness-related gene expression and physical proximity between REN-expressing tumor cells and natural killer (NK) cells. At spatial and single-cell resolution, REN-expressing tumor cells promoted NK cell exhaustion via PTN-NCL and COL4A1-CD44 ligand-receptor interactions, while showing limited impact on T cell dysfunction. Molecular docking identified estrogen-based compounds as potential REN inhibitors. Functional assays validated REN knockdown as significantly impairing tumor proliferation, migration, and survival *in vitro*.

**Discussion:**

This study establishes REN as a pivotal driver of tumor stemness and immune evasion in WT, playing a dual role in promoting tumor aggressiveness and suppressing NK-mediated immune surveillance. Targeting REN offers promising therapeutic opportunities for high-risk WT cases by simultaneously inhibiting tumor progression and restoring immune function. These findings emphasize REN’s potential as a transformative target for precision oncology and underscore the value of integrative transcriptomics in advancing personalized cancer treatment strategies.

## 1 Introduction

Wilms tumor (WT), the most common pediatric renal malignancy (1). Globally, malignant renal tumors account for approximately 5% of all cancers occurring before the age of 15. Each year, around 14,000 children (aged 0–14 years) are diagnosed with renal tumors worldwide, with regional variations in mortality. The incidence of WT varies between regions and ethnicities (2, 3). Despite advancements in multimodal therapies, including nephrectomy, radiation, and anthracycline-based chemotherapy, 15–20% of patients develop relapsed or refractory disease (4). These cases are often associated with chemotherapy resistance (5) and long-term cardiopulmonary toxicity (6). Current risk stratification protocols primarily rely on histopathological features and staging; however, they fail to account for the molecular heterogeneity within tumors (7), leading to suboptimal outcomes for high-risk patient subsets. Interestingly, nephrogenic rests—clusters of embryonic renal precursor cells—are detected as premalignant lesions in 30–40% of WT cases (8). This observation highlights the urgent need for molecular markers capable of predicting malignant transformation, as well as improved therapeutic strategies aimed at precision interventions.

The cancer stem cell (CSC) paradigm has significantly reshaped our understanding of tumor biology, demonstrating that a specialized subset of cells with self-renewal and differentiation capacities serves as the driving force behind tumor initiation, chemoresistance, and metastasis across diverse cancer types (9–11). Stemness indices derived from genome-wide multi-omics studies reveal that tumor stemness correlates with advanced disease states and therapeutic failure across multiple malignancies (12, 13). In WT, nephrogenic progenitor cells co-expressing SIX2 and CITED1 have been identified as tumor-initiating cells, capable of recapitulating tumorigenesis in xenotransplantation models (11). Spatial transcriptomics analyses suggest that these progenitor populations dynamically regulate self-renewal versus differentiation through integrin β1/β4 signaling pathways (11), revealing deeply conserved mechanisms underlying renal development and tumor progression.

Emerging evidence underscores tumor stemness as a pivotal regulator of the tumor immunosuppressive microenvironment (TIME). CSCs employ diverse mechanisms to modulate immune evasion, including secretion of cytokines that recruit myeloid-derived suppressor cells (MDSCs) and regulatory T cells (Tregs) (14), upregulation of immune checkpoint molecules such as PD-L1 and CTLA-4 (15), and suppression of natural killer (NK) cell proliferation and cytotoxic activity through membrane-bound TGF-β and PGE2 signaling (16, 17). Additionally, CSCs directly downregulate the expression of activating receptors on NK cells, including NKp44, NKp30, NKG2D, and DNAM-1, while promoting a phenotypic shift of NK cells toward the CD56dim subset. As CD56dim NK cells exhibit diminished degranulation capacity, this phenotypic shift directly attenuates their cytotoxic responses against cancer cells (14, 16, 17). Collectively, these mechanisms establish an immune-privileged niche that supports CSC survival within the TIME.

Although significant progress has been made in mapping CSC-mediated immune dynamics in adult cancers (18), the pediatric-specific implications regarding WT remain poorly defined. A critical knowledge gap persists in elucidating how WT-specific CSC regulators orchestrate immune evasion programs (1, 11). While SIX2+CITED1+ WT progenitor cells have been demonstrated to drive tumor initiation (11), the molecular triggers enabling their survival and immune evasion within the immunocompetent microenvironment of WT remain elusive. Understanding these regulatory mechanism is essential for advancing therapeutic strategies that can disrupt CSC-mediated immune evasion and reduce tumor progression.

In this study, a comprehensive multi-omics approach was employed, integrating single-cell RNA sequencing (scRNA-seq), spatial transcriptomics, and bulk RNA-seq data, alongside advanced machine learning algorithms, to unravel the complex interactions between REN-mediated tumor stemness and NK cell immune evasion in Wilms tumor. Our findings highlight REN as a critical regulator of CSC maintenance and immune suppression, positioning it as a promising therapeutic target for the treatment of high-risk WT. This integrative analysis provides a foundation for identifying novel intervention strategies that may attenuate tumor progression and improve clinical outcomes in WT patients.

## 2 Materials and methods

### 2.1 Comprehensive transcriptomics data acquisition and preprocessing

To enable an in-depth and multifaceted analysis of Wilms tumor (WT), this study incorporates a range of high-dimensional omics datasets, including bulk RNA sequencing (RNA-seq), single-cell RNA sequencing (scRNA-seq), and spatial transcriptomics data. The RNA-seq data and associated clinical information for the WT cohort were sourced from the UCSC Xena database (https://xena.ucsc.edu/) using the GDC TARGET-WT dataset. For downstream analyses, raw gene expression data were normalized to transcripts per million (TPM) values, and patients with an overall survival time shorter than 30 days were excluded to ensure robust analysis. Additionally, the GSE11024, GSE66045, and GSE73209 datasets were obtained from the GEO (https://www.ncbi.nlm.nih.gov/geo/) database and utilized for validating key genes at the bulk transcriptomic level.

A high-quality single-cell RNA-seq dataset from a pediatric WT patient (aged 84 months) was retrieved under the GEO accession number GSM53443671 (11). Raw sequencing data were aligned and quantified using STAR software with the GRCh38 reference genome. Additional single-cell RNA-seq datasets from three WT patients (aged 19, 27, and 57 months) were obtained from Young et al.’s study (19), forming a comprehensive set for cellular heterogeneity assessment.

To further evaluate the spatial architecture of WT, spatial transcriptomic data were accessed through the ScPCA Portal (https://scpca.alexslemonade.org/). These data included 10X Visium spatial transcriptomic data representing favorable and anaplastic histological classifications, enabling spatially resolved gene expression analyses critical for understanding tumor microenvironment heterogeneity. To elucidate candidate gene dependencies and effects within this, the cancer cell lines data was obtained from the Dependency Map (DepMap, https://depmap.org/portal/) portal.

### 2.2 Quantification and identification of tumor stemness signatures in Wilms tumor

Tumor stemness indices were quantified using the one-class logistic regression (OCLR) machine-learning algorithm (12), which has been widely adopted for stemness-related studies. The mRNA stemness index (mRNAsi) for each WT sample was computed by integrating gene expression profiles with the stemness signatures weighted by the model coefficients. This approach provided a quantitative measure of stemness across samples within the TARGET-WT cohort. To identify WT-specific tumor stemness-associated genes, differential expression analysis was conducted by stratifying samples based on the median mRNAsi value. Key genes were selected as WT-associated stemness markers based on the criteria of log fold change (logFC) > 0.5 and adjusted p-value < 0.05.

### 2.3 Tumor microenvironment evaluation in relation to stemness indices

To comprehensively characterize the tumor microenvironment (TME) in WT, the “IOBR” R package was utilized, implementing eight distinct immune infiltration algorithms. This multi-dimensional approach enabled a robust evaluation of immune cell populations and their activity within the TME. Spearman correlation analysis was performed to investigate the relationships between the mRNAsi and various immune cell populations, as well as key TME indicators. To further explore the functional implications of tumor stemness in modulating the TME, tumor signature scores and TME scores were calculated and compared across subgroups stratified by mRNAsi levels.

### 2.4 Development of cancer stemness prognostic index using integrated machine learning algorithms

To systematically develop a robust Cancer Stemness Prognostic Index (CSPI) for WT, we utilized data from the TARGET-WT cohort, employing a five-fold cross-validation approach. Patients were randomly divided into training and testing datasets in a 6:4 ratio, ensuring balanced sampling for model construction and validation. The training cohort was utilized for feature selection, while the testing cohort and overall cohort were used for validation. Robust prognostic tumor stemness genes were identified using the LASSO (Least Absolute Shrinkage and Selection Operator) algorithm combined with the Bootstrap method, incorporating 10-fold cross-validation and 1,000 sampling replacements. Genes recurring more than 100 times across iterations were designated as candidate genes for CSPI construction (20). To develop the prognostic model, an ensemble of 101 integrated machine learning algorithms was implemented in the training cohort. These algorithms included Random Survival Forest (RSF), Elastic Network (Enet), LASSO regression, Ridge regression, stepwise Cox regression, CoxBoost regression, partial least squares regression for Cox (plsRcox), Supervised Principal Components (SuperPC), generalized boosted regression modeling (GBM), and Survival Support Vector Machine (survival-SVM), all within a leave-one-out cross-validation (LOOCV) framework (21). The optimal model was selected based on the highest average concordance index (C-index), a metric reflecting the predictive accuracy of survival outcomes. To objectively assess its prognostic predictive performance, 1-, 3-, and 5-year calibration curves, time-dependent receiver operating characteristic (ROC) analyses, and Kaplan-Meier (KM) survival analyses were conducted. The optimal cutoff value for stratifying risk groups was determined algorithmically within the training cohort to minimize subjective bias. This cutoff was subsequently applied to the testing cohort and overall dataset to ensure consistent risk group classification. Finally, univariate and multivariate Cox regression analyses were performed to evaluate the prognostic robustness and efficiency of the CSPI in conjunction with various clinical variables.

### 2.5 Pathway enrichment analysis

To explore pathway-level distinctions between high- and low-CSPI groups, tumor hallmark pathway activity was assessed using the “GSVA” R package. Patients were grouped based on the median CSPI score, allowing systematic comparisons of functional pathway activity. Subsequently, differential pathway activity between the groups was evaluated using the “limma” R package, with hallmark pathways exhibiting an absolute t-value > 1 defined as significantly enriched or differentiated pathways. Further KEGG pathway enrichment analysis was performed using the “clusterProfiler” R package to identify potential signaling pathways enriched across different functional categories. This analysis was based on differentially expressed genes (DEGs) identified between the low- and high-CSPI groups.

### 2.6 Identification of prognostic tumor stemness genes

To pinpoint critical genes involved in tumor stemness within WT, a robust rank aggregation (RRA) algorithm was employed to perform a meta-analysis of DEGs across three independent transcriptomic datasets: GSE11024, GSE66045, and GSE73209. This method reduces dataset-specific biases and enhances the reliability of gene prioritization. DEGs were selected based on stringent criteria, including an absolute logFC > 0.5 and a p-value < 0.05, ensuring statistical significance and biological relevance. Following this, an intersection analysis was conducted between robust DEGs with the genes comprising the CSPI. This intersection highlighted the pivotal prognostic tumor stemness genes that potentially play essential roles in the tumorigenic and survival mechanisms of WT.

### 2.7 Comprehensive single-cell analysis of the tumor microenvironment in Wilms tumor

To elucidate the TME and cellular heterogeneity in WT, scRNA-seq data from four WT patients were integrated for downstream analysis. A rigorous approach to minimize batch effects was employed, leveraging the Single-cell Integration Benchmarking (scIB) framework to balance the removal of technical artifacts while preserving biological signals. Nine widely-used methods for batch effect correction—scANVI, CellHint, FastMNN, BBKNN, Seurat v4 CCA, Combat, Harmony, scVI, and Scanorama—were systematically assessed. The method that achieved the highest overall scIB score was selected for subsequent analyses, reflecting its optimal performance in maintaining data integrity across samples and sequencing platforms. Cell annotation was conducted based on established marker genes derived from previous studies (19), ensuring accurate identification of cell types. Tissue-specific cellular preferences were evaluated using the observed/expected (O/E) ratio, providing insights into differential cell-type enrichment across tissues. To infer malignant potential, the “infercnvpy” Python package was implemented, using potentially malignant cell types from normal tissue as reference cells. Cellular clonality and malignancy were assessed using the “leiden” clustering algorithm, where clusters were stratified based on copy number variation (CNV) scores. Clusters with elevated CNV scores were classified as tumor cells, enabling a robust delineation of malignant populations within the TME. The “CellChat” R package was employed to systematically infer intercellular communication networks at the single-cell level, leveraging gene expression profiles to identify ligand-receptor interactions and deduce signaling pathways involved in cellular crosstalk (22).

### 2.8 Spatial transcriptomics analysis of the tumor microenvironment in Wilms tumor

To gain deeper insights into the spatial organization of the TME in WT, spatial transcriptomics (ST) data from tissues representing favorable and anaplastic histological subtypes were analyzed. Integrating spatial information with single-cell transcriptomic profiles, the “Cell2location” Python tool was utilized to deconvolute cell types and quantify their proportions across spatial spots. This approach leverages a deep learning framework to predict cell-type distribution based on scRNA-seq expression profiles and annotations, while the non-negative matrix factorization (NMF) algorithm was applied to identify patterns of cellular co-localization at the spatial level, highlighting spatial interactions between distinct cell populations (23). To further explore intercellular relationships within each spatial spot, the “MistyR” R package was employed. Using a machine learning model, this tool facilitates the prediction of cellular proximity and spatial dependencies by modeling interactions among neighboring cell types within localized regions (24). This spatial analysis provided a detailed characterization of histological differences and cellular spatial dynamics, offering critical insights into the structural organization and functional interplay within the WT TME.

### 2.9 Molecular docking analysis

Molecular docking analysis was conducted to identify potential interactions between REN and candidate ligands. The Comparative Toxicogenomics Database (CTD; https://ctdbase.org/) under the “Kidney Neoplasms” category was used to explore potential drugs targeting REN and their effects on REN mRNA and protein expression. The protein structure of REN was obtained from the Protein Data Bank (PDB; https://www.rcsb.org/), while the chemical structures of candidate drugs were retrieved from PubChem database (https://pubchem.ncbi.nlm.nih.gov/). Docking simulations were performed using AutoDock Vina v1.2.2 to calculate binding affinity and molecular interaction patterns between ligands and REN. Docking results were considered significant if the binding energy was less than -5 kcal/mol and at least one hydrogen bond was formed between the ligand and the protein. This approach facilitated the identification of drugs with the potential to modulate REN function as a therapeutic target.

### 2.10 Cell culture and siRNA transfection

To investigate the functional role of REN in Wilms tumor cells, two cell lines were utilized: human embryonic kidney cells (293T) and Wilms tumor cells (WiT49). Both cell lines were obtained from the Cell Bank of the Chinese Academy of Sciences. The cells were maintained in Dulbecco’s Modified Eagle Medium (DMEM; Gibco), supplemented with 10% fetal bovine serum (FBS; Gibco) and 1% penicillin-streptomycin, under conditions of 37°C, 5% CO_2_, and high humidity. Regular sub-culturing was performed upon reaching 80% confluence to ensure optimal growth and viability throughout the experiments. To knock down REN expression in WiT49 cells, small interfering RNAs (siRNAs) targeting REN mRNA were designed and synthesized by Beijing Tsingke Biotech Co., Ltd., along with complementary primer sequences for knockdown verification. Lipofectamine™ 3000 (Invitrogen) was utilized for siRNA transfection following the manufacturer’s instructions. Cells were seeded into 6-well plates at approximately 70% confluence, after which siRNA and transfection reagent complexes were prepared using Opti-MEM™ Reduced Serum Medium (Gibco) and subsequently applied to the cells. After transfection, cells were incubated for 48–72 hours to achieve effective silencing of REN. The success of REN knockdown was validated using reverse transcription quantitative PCR (RT-qPCR), ensuring robust and reliable gene silencing for downstream functional assays. Detailed siRNA and primer sequences are provided in **Supplementary Table 1**. Together, these experimental approaches established the foundation for subsequent analyses of REN’s role in Wilms tumor progression.

### 2.11 Cell functional assays

To evaluate the impact of REN silencing on cell behavior, several functional assays were conducted. Cell proliferation was measured using the Cell Counting Kit-8 (CCK-8) and EdU incorporation assays: WiT49 cells transfected with siRNA targeting REN or scrambled control siRNA were assessed at 24, 48, and 72 hours post-transfection, with EdU incorporation quantified via fluorescence microscopy. Migration ability was determined using a wound healing assay, where monolayer scratches were monitored at 0, 12, and 24 hours for closure rates. Transwell assays were used to assess migration and invasion capabilities; cells seeded in serum-free DMEM migrated through transwell inserts toward 10% FBS in the lower chamber, with Matrigel pre-coating for invasion assays. Apoptotic populations were quantified using flow cytometry, where Annexin V-FITC/PI staining highlighted early and late apoptotic cells. Finally, cell cycle distribution was analyzed by staining transfected cells with propidium iodide (PI) after ethanol fixation, followed by flow cytometric quantification of cells in G1, S, and G2/M phases. Each assay provided detailed insights into the functional roles of REN in Wilms tumor cell physiology.

### 2.12 Statistical analysis

The processing and statistical analysis of transcriptome data were performed using R software (Version 4.3.2). Log-rank tests and Kaplan–Meier survival analyses were employed to assess the prognostic impact of genes within the TARGET-WT cohort. Experimental data were processed using GraphPad Prism (Version 9.4.1), and group comparisons were conducted using appropriate statistical methods, including t-tests, one-way analysis of variance. All experiments were conducted in triplicate or more. A p-value < 0.05 was deemed statistically significant in this study.

## 3 Results

### 3.1 Elevated tumor stemness drives Wilms tumor progression

**Figure 1:**
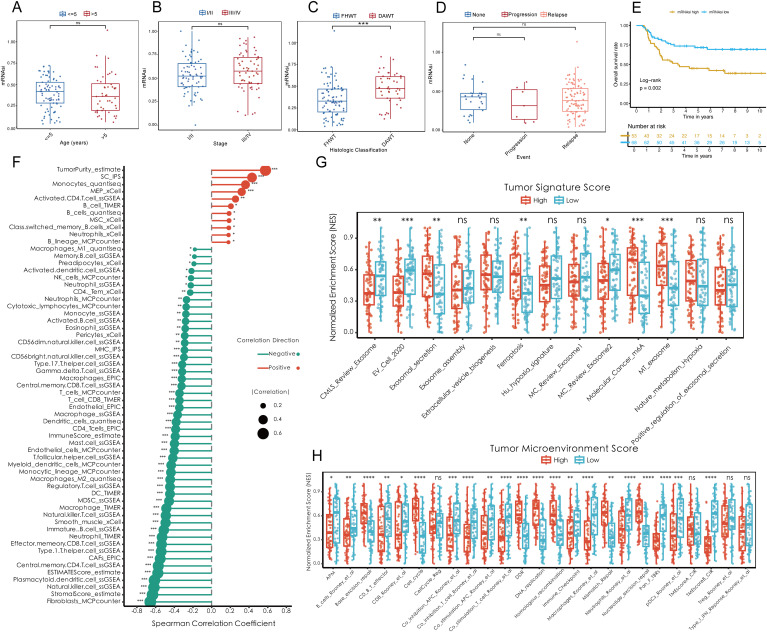
Elevated tumor stemness promotes Wilms tumor progression and shapes the tumor microenvironment. **(A–D)** Comparison of mRNAsi across clinical and pathological parameters, including age **(A)**, tumor stage **(B)**, histologic classification **(C)**, and tumor event status **(D)**, within the TARGET-WT cohort. **(E)** Kaplan-Meier survival analysis demonstrating the association between mRNAsi levels and overall survival. **(F)** Spearman correlation analysis between mRNAsi and tumor microenvironment components, including tumor purity, suppressor cell populations, and immune cell infiltration. **(G)** Tumor-specific signature scores highlighting molecular processes enriched in high mRNAsi groups, including exosome assembly, ferroptosis, and m6A methylation. **(H)** Evaluation of tumor microenvironment scores stratified by mRNAsi, showing alterations in oncogenic pathways such as excision repair, CD8+ effector cell abundance, and cell cycle regulation. Statistical significance was assessed using appropriate tests, where *p < 0.05, **p < 0.01, ***p < 0.001, ****p < 0.0001, and ns denotes not significant.

To investigate the role of tumor stemness in Wilms tumor (WT) progression, various stemness metrics, including mDNAsi (DNA methylation-based stemness), mRNAsi (mRNA expression-based stemness index), DMPsi (differentially methylated probes-based stemness index), ENHsi (enhancer-based stemness index), EREG mDNAsi, and EREG mRNAsi, were calculated for each patient in the TARGET-WT cohort (**Supplementary Figure S1A**). The correlation analysis showed that mRNAsi unrelated with other stemness metrics as an independent regulator in WT (**Supplementary Figure S1B**). Comparative analysis demonstrated that mRNAsi was significantly elevated in diffuse anaplastic WT (DAWT) compared to favorable histology WT (FHWT), independent of patient age, tumor stage, or tumor event (**Figures 1A–D**). Survival analysis further revealed a strong association between elevated mRNAsi and reduced overall survival (OS), indicating that higher tumor stemness is closely linked to poor clinical outcomes (**Figure 1E**). Conversely, other tumor stemness metrics did not demonstrate a significant association with patient outcomes (**Supplementary Figures S1C–G**). On the other hand, mRNAsi, identified as a key independent stemness regulator, was subjected to downstream analyses to elucidate its functional role and mechanistic contributions within the TME. Correlation analysis underscored the interplay between mRNAsi and features of the TME. Specifically, mRNAsi showed a significant positive correlation with tumor purity and suppressor cell populations, while exhibiting a negative correlation with the infiltration of most immune cell types within the TME. High mRNAsi groups demonstrated elevated tumor-specific signature scores, encompassing key processes such as exosome assembly, ferroptosis, and m6A methylation, among others (**Figure 1G**). Additionally, analysis of TME scores highlighted the influence of mRNAsi on several oncogenic factors, including excision repair mechanisms, CD8+ effector cell abundance, cell cycle regulation and so on (**Figure 1H**). These findings collectively emphasize the pivotal role of tumor stemness in driving WT progression and shaping TME dynamics, providing valuable insights into potential therapeutic targets for improving patient outcomes.

### 3.2 Development of a superior prognostic index for cancer stemness using integrated machine learning

**Figure 2:**
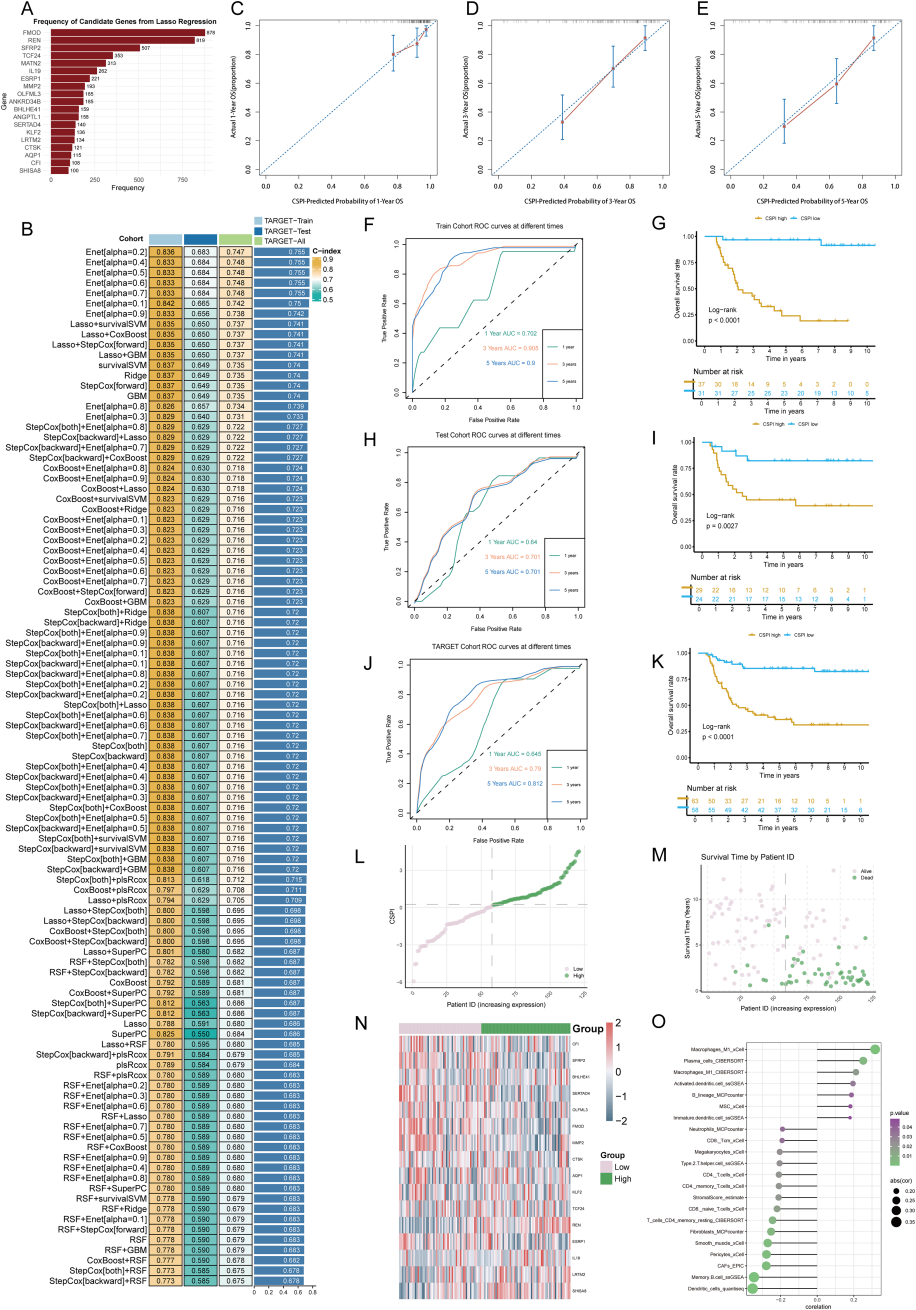
Development and validation of the cancer stemness prognostic index using integrated machine learning. **(A)** Frequency distribution of candidate genes identified through bootstrap-driven Lasso regression across 1,000 iterations. **(B)** Performance metrics (C-index) of CSPI derived from 101 integrated machine learning models, highlighting the superior predictive capability of the elastic net (Enet) method [α = 0.2]. **(C–E)** Calibration curves showing alignment between predicted probabilities and observed outcomes for 1-, 3-, and 5-year survival predictions, respectively. **(F, H, J)** Receiver operating characteristic (ROC) curves evaluating CSPI’s predictive performance in the training cohort **(F)**, test cohort **(H)**, and overall cohort **(J)**. **(G, I, K)** Kaplan-Meier survival curves demonstrating significantly worse clinical outcomes in patients with high CSPI scores across all cohorts. **(L, M)** Distribution and survival status of patients based on CSPI in the TARGET-WT cohort, stratifying individuals into high- and low-CSPI groups based on median CSPI values. **(N)** Heatmap of expression levels for 16 key genes constituting the CSPI across high and low CSPI groups. **(O)** Correlation analysis between CSPI scores and immune cell profiles.

**Figure 3:**
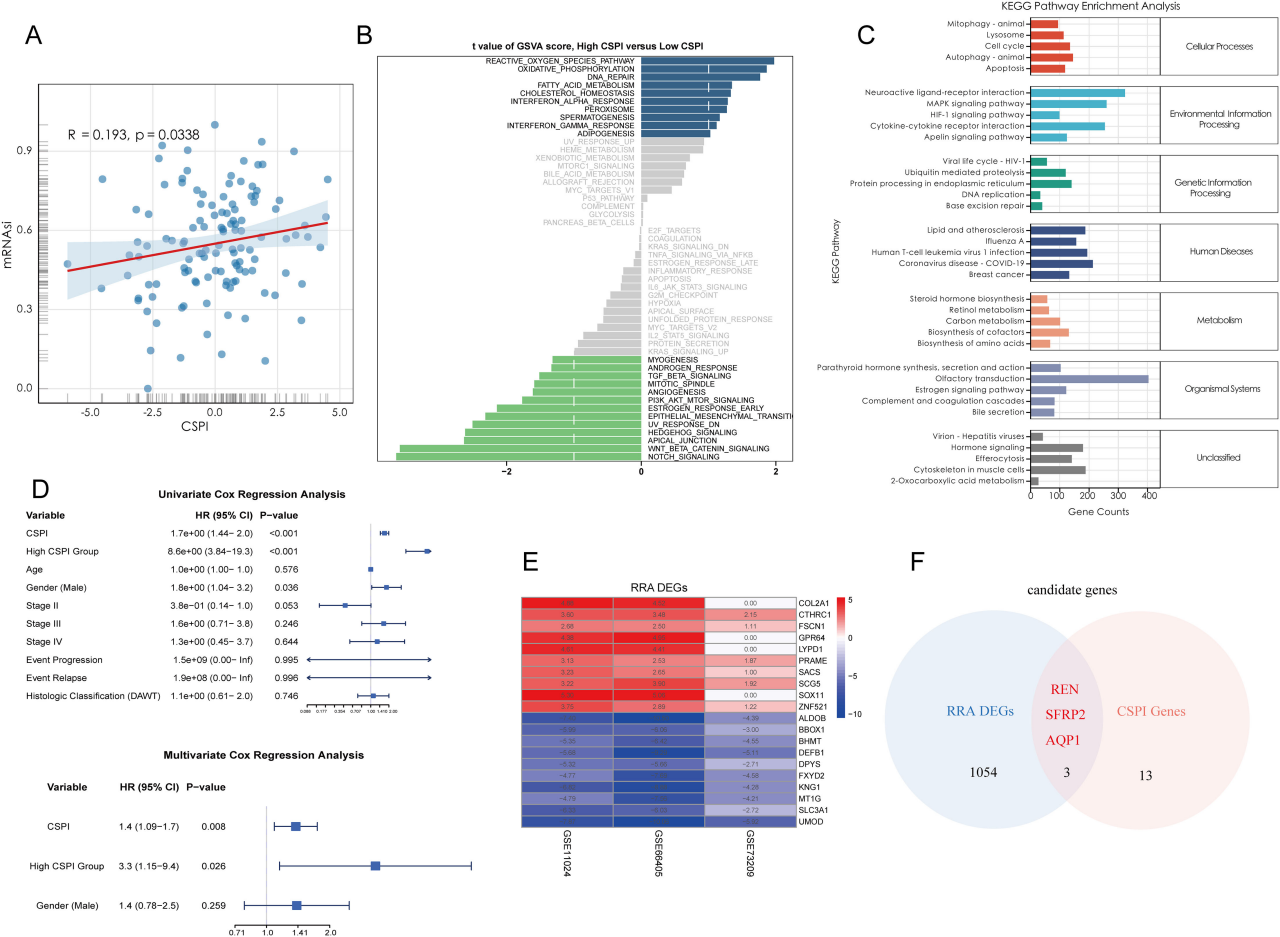
Functional characterization of CSPI and identification of core tumor stemness genes. **(A)** Correlation analysis demonstrating the positive association between CSPI scores and tumor stemness (mRNAsi), indicating elevated stemness in patients with high CSPI. **(B)** Gene Set Variation Analysis highlighting activation of key pathways in high-CSPI patients **(C)** KEGG pathway enrichment analysis of DEGs between high- and low-CSPI groups. **(D)** Univariate and multivariate Cox regression analyses identifying CSPI as an independent prognostic factor, emphasizing its robustness and predictive capability beyond clinical features. **(E)** Heatmap displaying the top 10 upregulated and downregulated DEGs between Wilms tumor tissues and adjacent normal tissues, derived from multi-cohort analysis using the RRA algorithm. **(F)** Venn diagram illustrating the intersection between RRA-selected DEGs and CSPI-associated genes, highlighting three core tumor stemness genes (REN, SFRP2, and AQP1).

To establish an efficient prognostic index for cancer stemness, a combination of machine learning approaches was employed. Using a bootstrap-driven Lasso regression algorithm, 19 key prognostic genes of tumor stemness were identified. These genes were selected for their high frequency (>100 occurrences) across 1,000 iterations in the training cohort (**Figure 2A**). Subsequently, 101 integrated machine learning algorithms were applied to construct the Cancer Stemness Prognostic Index (CSPI), which was validated across the test and overall cohorts. Among all tested models, the elastic net (Enet) method [α = 0.2] demonstrated superior predictive performance, achieving an impressive concordance index (C-index) of 0.755 (**Figure 2B**). The reliability and accuracy of CSPI predictions were supported by calibration curves, which demonstrated excellent alignment between predicted and observed outcomes (**Figures 2C, D**). Receiver operating characteristic (ROC) analyses further validated the model’s performance within each cohort. In the training cohort, CSPI achieved area under the curve (AUC) values of 0.702 for 1-year, 0.905 for 3-year, and 0.900 for 5-year survival predictions (**Figure 2F**). Comparable results were observed in the test cohort (AUC: 0.640, 0.701, and 0.701, respectively; **Figure 2H**) and the overall cohort (AUC: 0.645, 0.790, and 0.812, respectively; **Figure 2J**). Kaplan-Meier survival analyses revealed that patients with higher CSPI consistently exhibited worse clinical outcomes across all cohorts (**Figures 2G, I, K**). Classification of patients in the TARGET-WT cohort into high- and low-CSPI groups based on median CSPI values demonstrated a stark contrast in outcomes. The high CSPI group encompassed the majority of patients with fatal outcomes (**Figures 2L, M**). A heatmap illustrating the expression levels of 16 pivotal genes constituting the CSPI further emphasized their biological significance (**Figure 2N**). Correlation analysis with immune cell profiles revealed that high CSPI scores were associated with enhanced activation of M1 macrophages, accompanied by suppressed infiltration and activation of key immune cells such as CD4+ and CD8+ T cells (**Figure 2O**). In addition, correlation analysis indicated that patients in the high-CSPI group exhibited elevated tumor stemness (**Figure 3A**). Gene Set Variation Analysis (GSVA) showed that individuals with higher CSPI were characterized by the activation of pathways related to reactive oxygen species, oxidative phosphorylation, fatty acid metabolism, and DNA repair, elucidating the metabolic reprogramming observed in these patients with higher CSPI (**Figure 3B**). DEGs between the groups were significantly enriched in metabolic and oncogenic pathways, including carbon metabolism, amino acid biosynthesis, hypoxia-inducible factor 1 (HIF-1) signaling, and mitogen-activated protein kinase (MAPK) signaling pathways (**Figure 3C**). Further validation of CSPI’s prognostic robustness was achieved through univariate and multivariate Cox regression analyses, which identified CSPI as the sole independent prognostic factor when compared to various clinical features (**Figure 3D**). These findings underscore the robustness of CSPI as a novel predictive biomarker for cancer stemness-related outcomes and its potential to guide treatment strategies by linking stemness features with immune microenvironment dynamics.

### 3.3 Identification of core tumor stemness genes

DEGs between Wilms tumor tissues and adjacent normal tissues were systematically identified using the RRA algorithm applied to three independent datasets. This approach minimized dataset-specific biases and ensured a high-level integration of multi-cohort data. The resultant DEGs were ranked by their significance, and the top 10 upregulated and downregulated genes were visualized in a heatmap to highlight expression alterations that are consistent across datasets (**Figure 3E**). To further refine these findings, three pivotal tumor stemness-associated genes—REN, SFRP2, and AQP1—were selected for downstream analysis. These genes were prioritized based on their inclusion in the gene set constituting the CSPI (**Figure 3F**). This multi-layer filtering process underscores their biological significance and potential as therapeutic targets and diagnostic markers in stemness-driven oncogenesis.

### 3.4 Landscape of the tumor microenvironment in Wilms tumor

**Figure 4:**
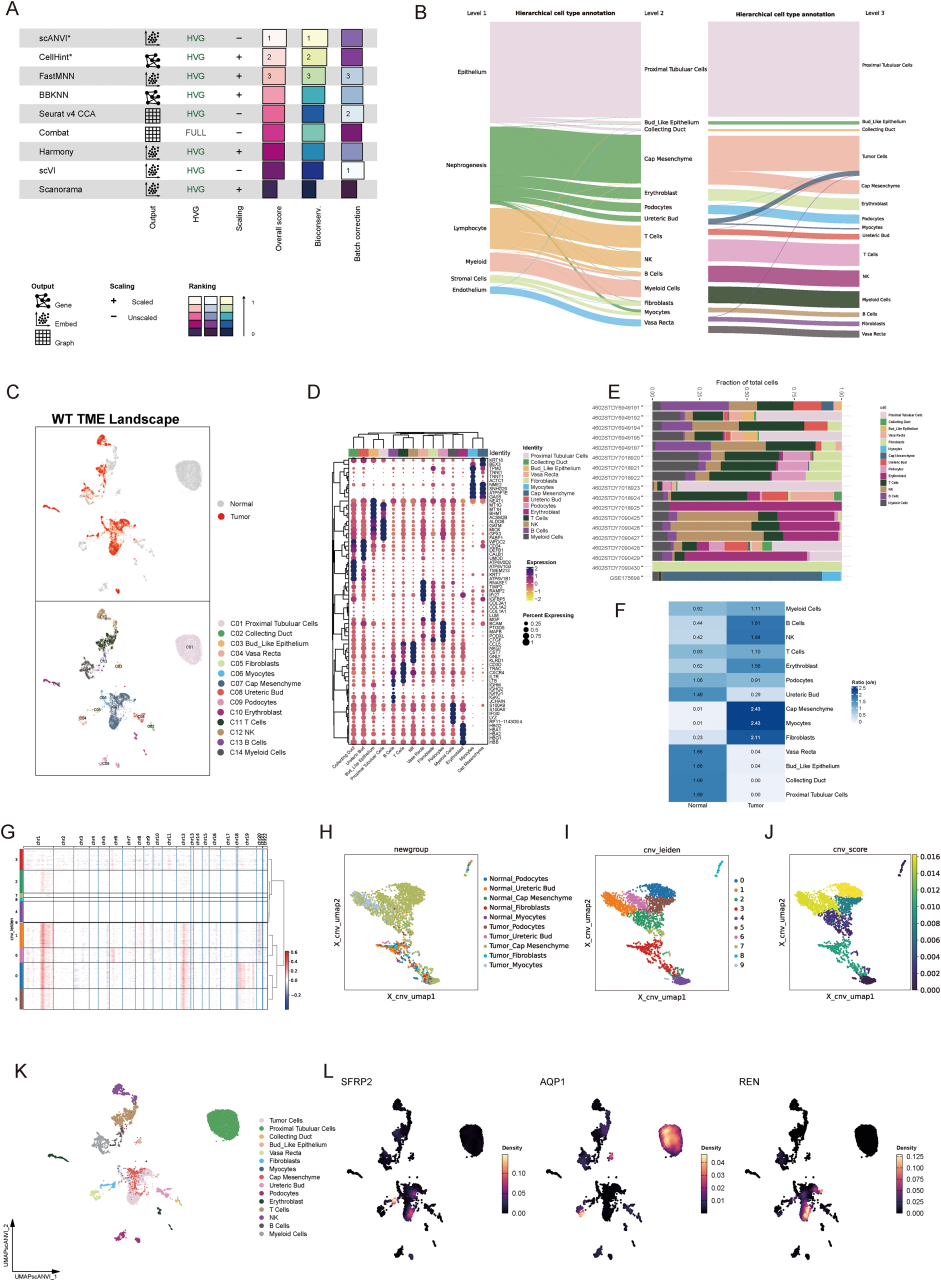
Single-cell landscape of the tumor microenvironment in Wilms tumor. **(A)** Evaluation of nine batch correction algorithms for scRNA-seq data processing, highlighting “scANVI” as the top-performing method for resolving batch effects while retaining biological variance. **(B)** Hierarchical cell annotation workflow illustrated via a Sankey plot, which depicts three levels of classification detailing cellular identities within the Wilms tumor microenvironment. **(C)** UMAP visualization of tissue group distribution and level 2 cell annotations, showcasing cellular heterogeneity across normal and tumor tissues. **(D)** Heatmap of cell type-specific marker gene expression, confirming accurate cell type identification and annotation. **(E)** Bar plot showing inter-sample variability in the proportion of identified cell populations, reflecting differences in cellular composition across individual samples. **(F)** Tissue preference analysis demonstrating significant enrichment of specific cell types within tumor tissues compared to normal tissues **(G)** Copy number variation analysis based on hierarchical clustering and CNV profiles, identifying clusters with malignant properties. **(H–J)** Visualization of CNV-based classification, including UMAP plots displaying CNV-derived clustering **(H)**, cell annotations incorporating CNV features **(I)**, and CNV scores distinguishing malignant cells **(J)**. **(K)** Updated level 3 cell annotations integrated with CNV data, pinpointing malignant tumor cell populations within the Wilms tumor microenvironment. **(L)** Validation of bulk RNA-seq-derived core stemness genes (SFRP2, AQP1, and REN) at single-cell resolution, revealing cell type-specific expression patterns.

To characterize the TME of Wilms tumor at a single-cell resolution, nine batch correction methodologies were assessed to enhance data quality. Among these, the “scANVI” algorithm demonstrated superior performance in eliminating batch-related artifacts while preserving biologically relevant variation, making it the preferred method for scRNA-seq data processing (**Figure 4A**). Cell annotation was performed using a hierarchical approach, as visualized in a Sankey plot illustrating three distinct levels of cell classification (**Figure 4B**). Uniform Manifold Approximation and Projection (UMAP) analysis displayed tissue group distribution and level 2 cell annotations, enabling an intuitive visualization of cellular heterogeneity (**Figure 4C**). Furthermore, a heatmap detailing the expression of marker genes for each cell type underscored the accuracy of cell type identification (**Figure 4D**). Notably, significant inter-sample variation was observed in cell population composition, highlighting differences in cellular architecture between samples (**Figure 4E**). Tissue preference analysis revealed that specific cell types, including cap mesenchyme, myocytes, fibroblasts, and immune cells, exhibited higher abundance within tumor tissues compared to non-tumor tissues, suggesting their active involvement in tumorigenesis (**Figure 4F**). To pinpoint malignant cells within the TME, CNV analysis, cell clustering, and hierarchical clustering were employed. These analyses utilized normal tissue-derived cells as reference points, enabling the identification of cell populations exhibiting amplified or deleted genomic regions and elevated CNV scores. Specifically, clusters 0, 1, 5, and 6 were identified as harboring malignant properties based on their CNV profiles, designating them as tumor cells (**Figures 4G–J**). Furthermore, various Wilms tumor cell marker genes were utilized to validate the annotation precision across all identified cell types. The results revealed that tumor cells exhibited significantly elevated expression levels of WT1, SIX1, SIX2, and CITED1, supporting the accurate annotation of tumor cells (**Supplementary Figure S1J**). Updated cell annotations at level 3 were subsequently integrated into the analysis for enhanced precision (**Figure 4K**). Finally, key genes derived from bulk RNA-seq analysis were validated at the single-cell level. This validation revealed cell type-specific expression patterns: SFRP2 was predominantly expressed in fibroblasts, AQP1 localized to proximal tubular cells, vasa recta, and bud-like epithelial cells, while REN was primarily expressed in tumor cells (**Figure 4L**).

### 3.5 Spatial validation of key tumor stemness gene expression

**Figure 5:**
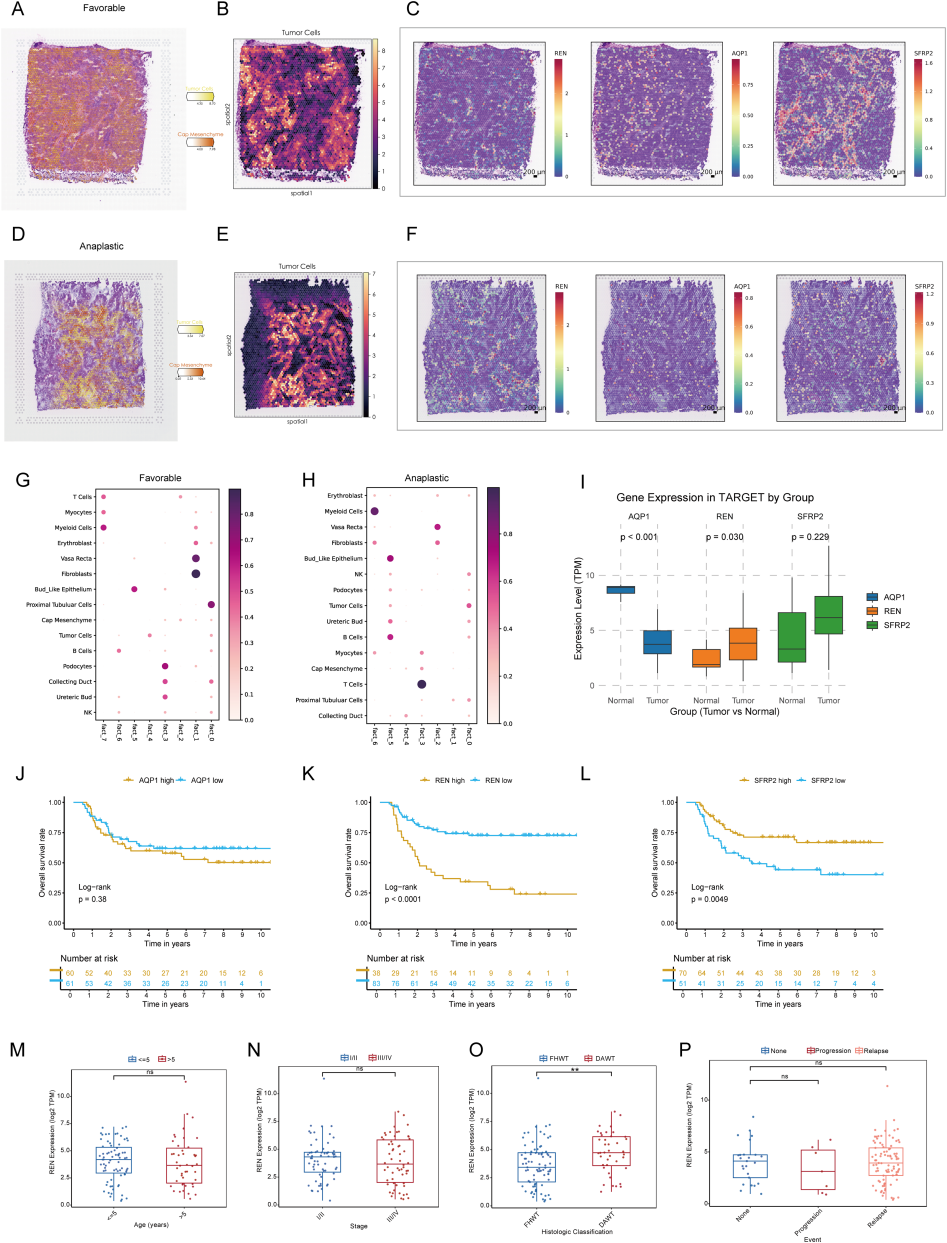
Spatial Transcriptomic Validation and Prognostic Analysis of Key Tumor Stemness Genes in Wilms Tumor. **(A–C)** Spatial deconvolution of favorable Wilms tumor tissues identifies tumor cells concentrated within specific clusters, indicative of high differentiation potential. REN demonstrates spatially restricted and tumor-specific expression, whereas SFRP2 and AQP1 show diffuse expression across the tissue. **(D–F)** Spatial deconvolution of anaplastic Wilms tumor tissues reveals diverse cellular co-localization, characteristic of lower differentiation and enhanced malignancy. REN maintains distinct tumor-specific localization, while SFRP2 and AQP1 display widespread expression patterns. **(G–H)** Cellular co-localization and interaction patterns analyzed using NMF algorithm. **(I)** Comparative analysis of gene expression in the TARGET-WT cohort between tumor and normal tissues, showing significant differential expression for REN and AQP1. **(J–L)** Kaplan-Meier survival analysis of AQP1, REN and SFRP2 in TARGET-WT cohort. **(M–P)** Comparison of REN expression across clinical and pathological parameters, including age **(M)**, tumor stage **(N)**, histologic classification **(O)**, and tumor event status **(P)**, within the TARGET-WT cohort. Statistical significance was assessed using appropriate tests, where **p < 0.01 and ns denotes not significant.

To integrate spatial transcriptomic data with scRNA-seq insights, spots from favorable and anaplastic Wilms tumor tissues were deconvoluted and mapped using scRNA-seq data. The top two most abundant cell populations for each spot were identified and visualized, highlighting cellular distribution across tumor regions (**Figures 5A, D**). The spatial expression patterns of three key tumor stemness genes—REN, SFRP2, and AQP1—were examined in both tumor subtypes. Notably, REN exhibited prominent localization within tumor regions, consistent with its characteristic expression at single-cell level. Conversely, SFRP2 and AQP1 were not confined to tumor regions, reaffirming their non-specific expression patterns observed in single-cell analysis (**Figures 5B, C, E, F**). These findings validate the single-cell data at a spatial resolution and confirm the functional and spatial distinctiveness of REN as a tumor-associated gene. To investigate cellular interactions and spatial co-localization patterns, the NMF algorithm was used to factorize spatial location profiles. Striking differences in cellular context were identified between favorable and anaplastic tumor subtypes. In favorable Wilms tumor tissue, tumor cells were primarily factorized into a single spatial cluster, indicative of their high differentiation characteristics and limited cellular interactions within the TME (**Figure 5G**). In contrast, anaplastic tumor tissue displayed a more diverse co-localization pattern; tumor cells were spatially associated with ureteric bud and podocyte populations—potential sources of malignant cells—reflecting lower differentiation and greater malignant potency. Moreover, an intriguing co-localization of tumor cells with NK and B cells was observed, suggesting potential immune modulation or evasion mechanisms within the anaplastic tumor subtype (**Figure 5H**). These results provide spatial-level validation of key stemness gene expression and reveal unique cellular interaction landscapes associated with different Wilms tumor histological subtypes.

### 3.6 REN as a key driver of Wilms tumor progression and prognostic marker

To investigate the role of pivotal tumor stemness genes in Wilms tumor progression, expression comparisons and Kaplan-Meier survival analyses were conducted using the TARGET-WT cohort. Among the three candidate genes—REN, SFRP2, and AQP1, only REN and AQP1 exhibited significant differential expression between tumor tissues and normal tissues (**Figure 5I**). However, survival analysis highlighted REN and SFRP2 as key prognostic markers impacting patient outcomes, with REN identified as the most critical regulator due to its consistent association with poor clinical outcomes across the cohort based on optimal cutoff values to avoid subjective bias and reveal prognostic value (**Figures 5J–L**). Further clinical subgroup analyses revealed no significant variation in REN expression among age groups, disease stages, histological classifications, or tumor event subtypes, except for its pronounced overexpression in DAWT. Higher REN expression was strongly correlated with malignant progression, tumor dedifferentiation, and increased aggressiveness (**Figures 5M–P**). This finding emphasizes the role of REN in driving Wilms tumor evolution toward the unfavorable DAWT subtype, which is associated with poorer clinical prognosis (**Figure 5O**). Subsequent expression analysis revealed that REN was significantly upregulated in the Higher-CSPI group (**Figure 2O** and **Supplementary Figure S1H**). Furthermore, REN expression showed a strong positive correlation with mRNAsi (**Supplementary Figure S1I**). Taken together, these findings identify REN as a key regulator of tumor stemness, warranting further downstream analyses to elucidate its mechanistic role.

**Figure 6:**
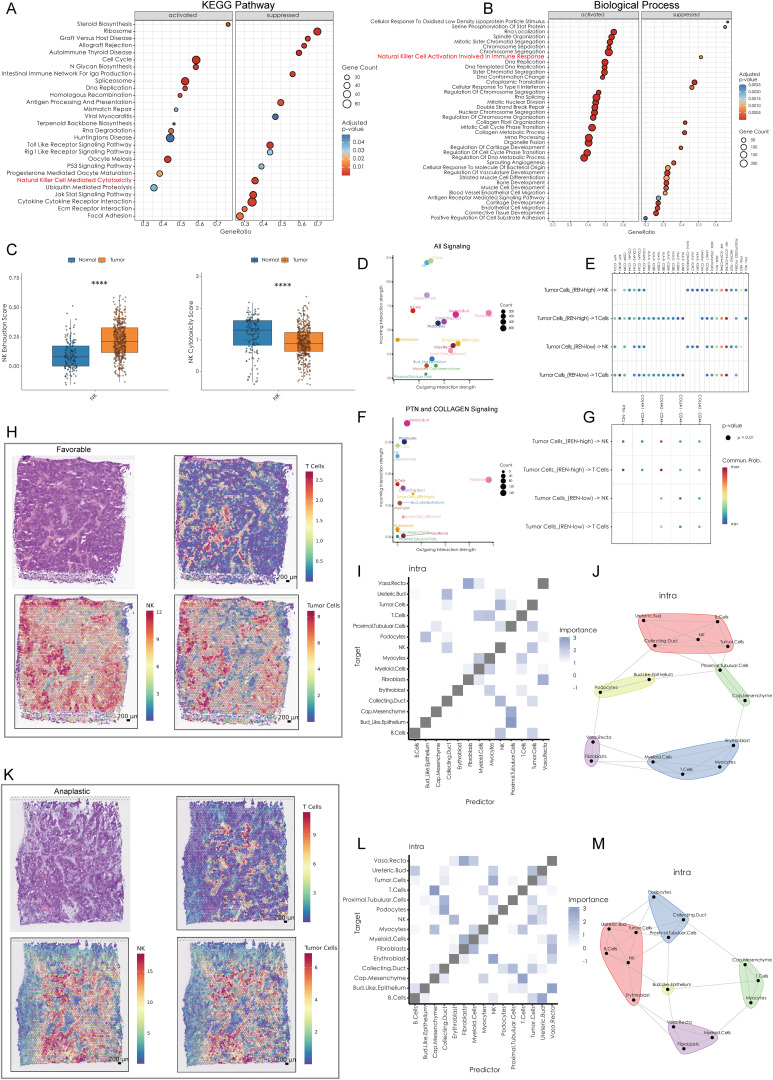
High REN Expression Reshapes Tumor-Immune Cell Communication and Spatial Dynamics in Wilms Tumors. **(A)** Gene Set Enrichment Analysis based on KEGG pathways, depicting the activated and suppressed biological pathways associated with REN expression in tumor tissues. **(B)** GSEA using Gene Ontology biological processes, highlighting the activated and suppressed biological processes associated with REN expression in tumor tissues. **(C)** Box plots illustrating NK cell functional impairment in tumor tissues, with reduced exhaustion markers and diminished cytotoxic activity compared to normal tissues. **(D)** Overall cell-cell communication network analysis within the TME, indicating NK and T cells as primary recipients of interaction signals from other cell types. **(E)** Comparison of cell-cell communication patterns in high-REN versus low-REN tumor cells. **(F)** PTN and COLLAGEN signaling networks reveal major producers and recipients within the TME. **(G)** Ligand-receptor pair analysis identifies REN-specific interactions, such as PTN-NCL and COL4A1-CD44, which are exclusively enriched in tumor cells with high REN expression and absent in low-REN-expressing cells. **(H, K)** Spatial maps illustrating tumor cell, NK cell, and T cell distributions across favorable and anaplastic subtypes of WT, highlighting physical proximity between tumor cells and NK cells. **(I, L)** Co-localization heatmaps exhibit the spatial relationship of each cell type in single spot across favorable and anaplastic subtypes of WT. **(J, M)** Co-localization network diagrams providing a detailed visualization of cell-to-cell co-localization preferences within the TME. Statistical significance was assessed using appropriate tests, where ****p < 0.0001, and ns denotes not significant.

To unravel the molecular mechanisms underlying REN-mediated tumor progression, Gene Set Enrichment Analysis (GSEA) was performed. The results revealed that REN profoundly activates multiple oncogenic pathways, including cell cycle regulation, DNA replication, and homologous recombination, while simultaneously suppressing the P53 signaling pathway and immune functions, specifically those mediated by NK cells (**Figure 6A**). Furthermore, functional enrichment analyses confirmed that REN is implicated in biological processes such as immune modulation and suppression of NK cell activation, which plays a critical role in anti-tumor immunity (**Figure 6B**). Collectively, these findings underscore REN as a key regulator of tumor stemness and a potent oncogene in Wilms tumor. Beyond driving tumor progression, REN functions as an immune suppressor, particularly by impairing NK cell-mediated tumor surveillance mechanisms. The identification of REN as both a prognostic marker for poorer outcomes and a critical driver of oncogenesis suggests its potential as a therapeutic target for combating stemness-driven Wilms tumor progression and immune evasion.

### 3.7 REN drives tumor cell evasion of NK cell-mediated immune surveillance

A comprehensive analysis was performed to elucidate the mechanisms by which WT cells evade immune killing by NK cells. scRNA-seq data was utilized to investigate cellular interactions at the single-cell level, while ST data validated spatial proximity and interaction feasibility across the TME. The scRNA-seq data revealed that NK cells within the WT TME exhibit significant immune exhaustion, characterized by reduced immune cytotoxicity and functional impairment (**Figure 6C**). Furthermore, NK cells and T cells emerged as the top two cell types receiving outgoing interaction signals from other cell types within the TME, indicating their central roles in immune-tumor dynamics (**Figure 6D**). Notably, tumor cells with high REN expression exhibited distinct differences in cellular communication patterns with NK and T cells, especially through PTN and COLLAGEN signaling pathways (**Figure 6E**). In the context of PTN and COLLAGEN signaling, both NK and T cells were major recipients of these outgoing signals, while fibroblasts and high-REN-expressing tumor cells acted as major producers of the signaling molecules (**Figure 6F**). Ligand-receptor (L-R) pair analyses indicated that tumor cells with high REN expression engaged NK and T cells through specific ligand-receptor pairs—namely PTN-NCL and COL4A1-CD44, interactions that were largely absent in low-REN-expressing tumor cells (**Figure 6G**).

Spatial analyses further validated these cellular interactions. The spatial distribution of tumor cells, NK cells, and T cells was analyzed in both favorable and anaplastic WT tissues. Tumor cells and NK cells were shown to share similar spatial distributions in both tumor subtypes, suggesting frequent physical proximity and interaction (**Figures 6H, K**). Spatial co-localization analysis revealed that tumor cells co-localize significantly with NK cells, both as predictors and targets, within individual ST spots. However, this relationship was not observed between tumor cells and T cells, or between fibroblasts and NK/T cells (**Figures 6I, L**). Detailed cell co-localization patterns for both WT subtypes were visualized in diagrams, highlighting tumor cells’ preferential interactions with NK cells over other immune cell types (**Figures 6J, M**). These findings suggest that high-REN-expressing tumor cells actively manipulate cellular interactions to evade NK cell-mediated immune surveillance. By leveraging specific ligand-receptor interactions, such as PTN-NCL and COL4A1-CD44, tumor cells impose a regulatory influence on NK cells while bypassing significant engagement with T cells or fibroblasts. This indicates that REN serves as a central regulator of NK cell immune evasion in the WT TME, offering potential avenues for targeted therapeutic intervention to restore NK cell functionality and enhance anti-tumor immunity.

### 3.8 REN promotes tumor proliferation, migration, and invasion in Wilms tumor cells

**Figure 7:**
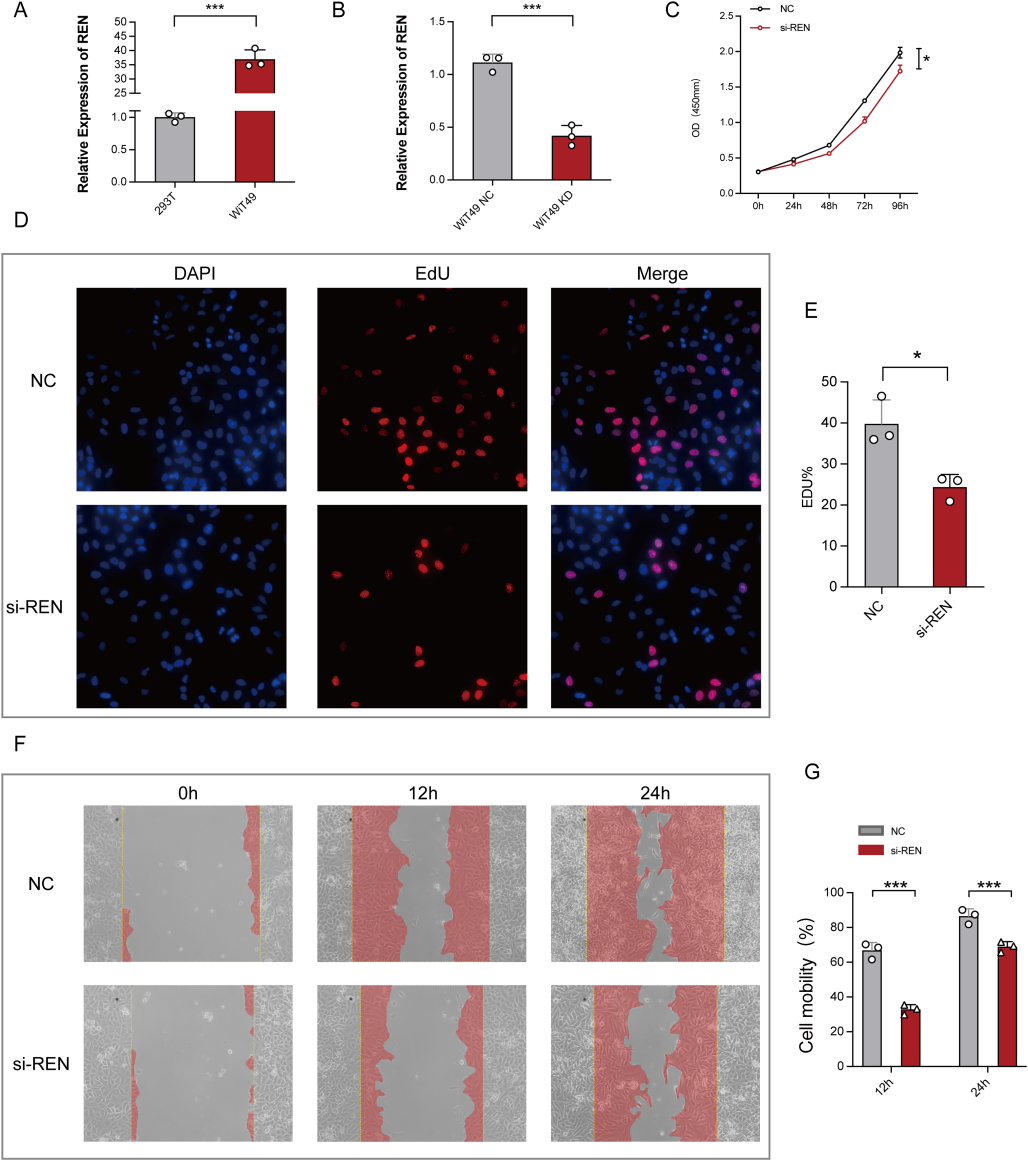
Functional Validation of REN Expression in Wilms Tumor Cells. **(A)** Relative REN mRNA expression levels as analyzed by RT-qPCR, demonstrating significant upregulation of REN in WiT49 cells compared to 293T cells. **(B)** Efficiency of REN knockdown using siRNA in WiT49 cells, as confirmed by RT-qPCR, showing significantly reduced REN expression compared to the negative control (NC) group. **(C)** CCK-8 proliferation assay assessing cellular activity over time, showing reduced proliferation rates in REN-knockdown (si-REN) cells compared to the NC group. **(D)** Fluorescent images from EdU incorporation assays comparing DNA synthesis in NC and si-REN groups. Representative images show DAPI staining (nuclei), EdU incorporation (cell proliferation), and merged overlays. **(E)** Quantification of EdU-positive cells indicates a significant reduction in tumor cell proliferation in the si-REN group compared to the NC group. **(F)** Representative images of wound healing assays evaluating cell migration at 0, 12, and 24 hours in NC and si-REN groups. Red shading denotes migration area. **(G)** Quantitative analysis of cell mobility at 24 and 48 hours post-wound generation, showing significantly lower migration rates in si-REN cells compared to NC cells. Statistical significance was assessed using appropriate tests, where *p < 0.05, ***p < 0.001 and ns denotes not significant.

**Figure 8:**
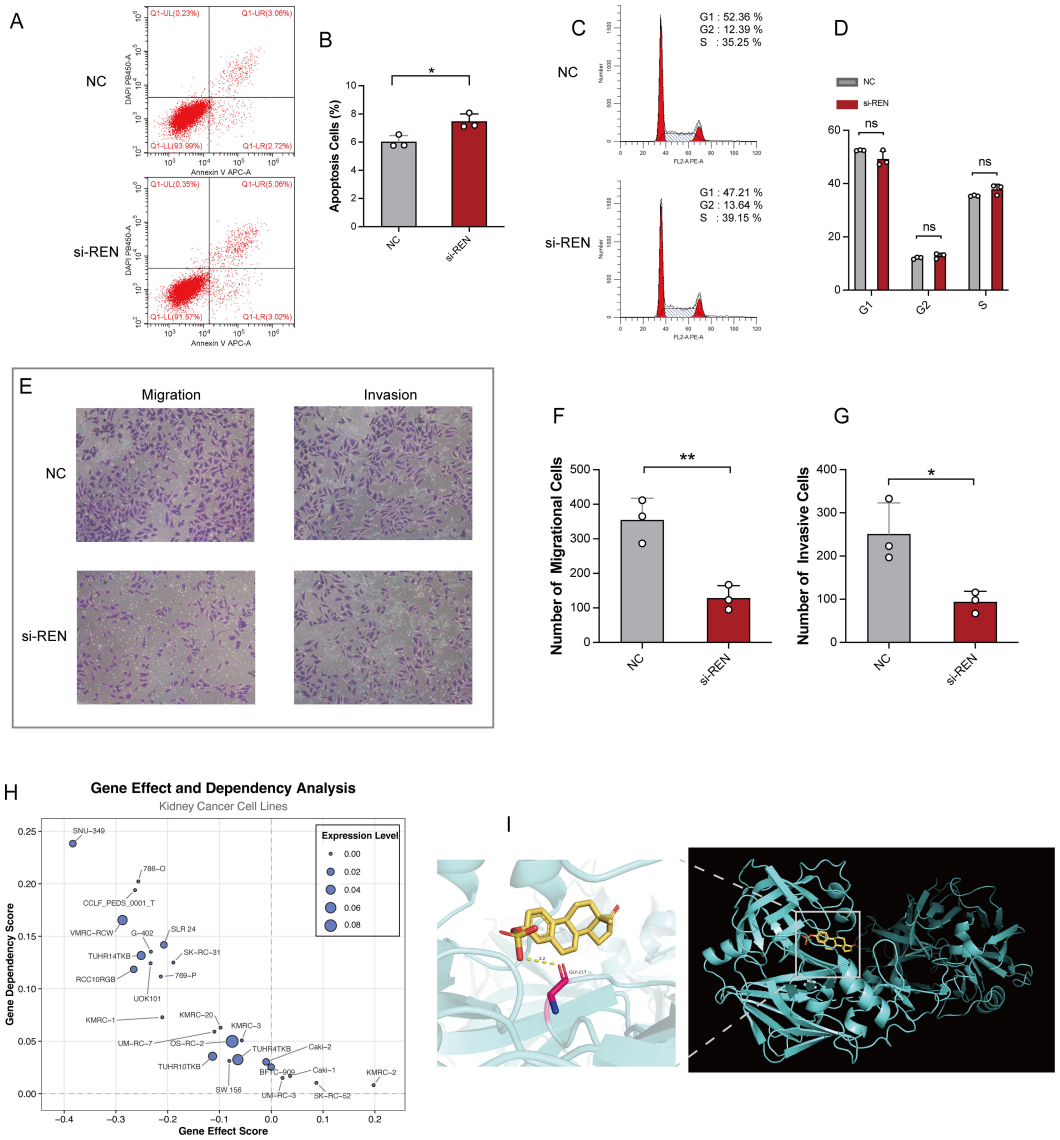
REN Knockdown Enhances Apoptosis and Impairs Tumor Migration, Invasion, and Survival. **(A)** Representative flow cytometry plots showing apoptosis rates in the REN-knockdown (si-REN) and negative control (NC) groups. **(B)** Quantification of apoptotic cells, revealing significantly higher apoptosis rates in the si-REN group compared to the NC group. **(C)** Flow cytometry analysis of cell cycle distribution, presenting G1, G2, and S phase percentages in both NC and si-REN groups. **(D)** Statistical comparison of cell cycle distribution, showing no significant differences between the NC and si-REN groups across all phases. **(E)** Representative images of transwell migration and invasion assays comparing NC and si-REN cells. **(F)** Quantitative analysis of migratory cells, showing a significant reduction in cell migration upon REN knockdown. **(G)** Quantification of invasive cells, indicating a significant decrease in invasive capability in the si-REN group compared to the NC group. **(H)** Gene dependency analysis using DepMap data for 26 renal carcinoma cell lines, illustrating that most kidney cancer cell lines exhibit high dependency on REN expression for survival. **(I)** Molecular docking model showing the interaction between estrogen proteins and the REN protein, with predictions indicating binding events that lead to reduced REN mRNA expression. Statistical significance was assessed using appropriate tests, where *p < 0.05, **p < 0.01 and ns denotes not significant.

Functional studies were conducted to investigate REN’s role in Wilms tumor progression using Wilms tumor cell line WiT49 and human embryonic kidney cell line 293T as a control. REN mRNA expression was validated via RT-qPCR, revealing significant upregulation in WiT49 cells compared to 293T cells (**Figure 7A**). To assess its function, REN expression in WiT49 cells was knocked down using small interfering RNA (siRNA), resulting in significantly reduced REN expression levels as confirmed by RT-qPCR (**Figure 7B**). Functional assays demonstrated the tumor-promoting role of REN in Wilms tumor cells. A CCK-8 proliferation assay revealed a significant reduction in cellular activity and proliferation in the REN-knockdown (KD) group compared to the negative control (NC) group (**Figure 7C**). Further validation using the EdU incorporation assay showed markedly slower rates of tumor cell proliferation in the KD group, highlighting REN’s oncogenic influence on cellular growth (**Figures 7D, E**). Migration ability was assessed through wound healing assays, which demonstrated significantly reduced migration of tumor cells in the KD group at both 12-hour and 24-hour time points (**Figures 7F, G**). Apoptosis and survival analyses confirmed that REN contributes to tumor cell viability. Flow cytometry-based apoptosis assays revealed higher apoptosis rates in the KD group, indicating that REN supports tumor cell survival mechanisms (**Figures 8A, B**). Interestingly, cell cycle distribution analysis found no significant differences between the KD and NC groups in the G1, G2, or S phases (**Figures 8C, D**), suggesting that REN may influence tumor progression independently of cell cycle regulation. Invasion and migration capabilities were further assessed using transwell assays, which demonstrated a significant reduction in the number of migratory and invasive cells following REN knockdown (**Figures 8E–G**). This suggests that REN plays a critical role in facilitating tumor cell invasiveness. Knockout data from the DepMap database supported this finding, showing that most renal carcinoma cell lines, including clear cell renal cell carcinoma and other subtypes, exhibit high dependence on REN for survival, with cell death or reduced proliferation observed after REN knockout across 26 kidney cancer cell lines (**Figure 8H**). Molecular docking analysis provided deeper mechanistic insight, revealing that estrogen proteins can effectively bind to the REN protein and reduce REN mRNA expression (**Figure 8I**). This interaction suggests potential therapeutic strategies targeting REN regulation via estrogen-based compounds to mitigate tumor progression. Together, these findings highlight REN as a critical driver of Wilms tumor cell proliferation, survival, migration, and invasion. The dependence of tumor cells, particularly renal carcinoma cell models, on REN underscores its potential as a therapeutic target. Future work should focus on developing targeted approaches to inhibit REN and disrupt its oncogenic functions, particularly in renal cancer subtypes such as Wilms tumor.

## 4 Discussion

Wilms tumor (WT), the most common renal malignancy in infants and young children, is closely tied to aberrations in early kidney development. While current multimodal therapies—comprising surgery, chemotherapy, and radiotherapy—achieve cure rates exceeding 90% in most cases, 20% of patients experience relapse, and 25% of long-term survivors report severe complications, including renal failure, cardiovascular disorders, and secondary malignancies (3). These factors underscore the urgent need to unravel the molecular mechanisms underlying WT, enhance prognostic accuracy, and develop more refined therapeutic strategies. Tumor stemness, a hallmark characteristic of cancer stem cells (CSCs), plays a critical role in tumorigenesis, progression, relapse, and resistance to therapy (11). WT, in particular, has been shown to heavily rely on tumor stemness for its aggressive behavior and resistance to conventional treatments (12).

In this study, we employed a multi-omics integrative approach, combining bulk RNA-seq and scRNA-seq datasets with advanced machine learning techniques, to unravel the molecular mechanisms underlying tumor stemness in WT. By leveraging integrated machine learning methodologies, we successfully developed the Cancer Stemness Prognostic Index (CSPI), a robust predictive model with exceptionally high prognostic accuracy. The CSPI effectively stratified patients into distinct risk groups, demonstrating that those with higher CSPI scores exhibited elevated tumor stemness and a significantly poorer clinical outcome. Our findings further revealed that patients with high CSPI scores exhibited notable metabolic reprogramming, characterized by the upregulation of pathways related to reactive oxygen species (ROS) metabolism and oxidative phosphorylation. These metabolic shifts appear to play a critical role in sustaining tumor stemness and driving tumor progression (25). Conversely, patients within the low CSPI group primarily relied on canonical stemness-associated signaling pathways, including the Wnt and Notch pathways, as previously reported (9, 13). This dichotomy highlights the heterogeneous mechanistic strategies that WT tumors employ to maintain their stemness and underscores the importance of tailoring therapeutic approaches accordingly. After comprehensive and robust multidimensional validation, incorporating differential expression analyses and survival assessments, three key tumor stemness-associated genes—REN, AQP1, and SFRP2—were carefully compared. Of these, REN was the only gene exhibiting significantly elevated expression in WT tissues and demonstrating a profound impact on patient outcomes. Further scRNA-seq and ST analyses revealed enrichment of REN expression specifically within tumor cells and tumor regions, underscoring its spatial and cellular relevance in Wilms tumor biology. Given these findings, REN was identified as a critical regulator of tumor stemness in WT, warranting further investigation into its functional roles and potential mechanisms within the TME. Moreover, integrated transcriptome approach revealed REN as a critical regulator of tumor stemness and a major contributor to NK cell immune evasion. REN was found to be overexpressed in WT, correlating strongly with poor survival outcomes and advancing CSC-like properties by enhancing stemness and impairing differentiation potential, thereby potentiating tumor aggression, particularly in the DAWT subtype. Intriguingly, REN transcription has been shown to be regulated by the WT1 gene (specifically the WT1-KTS isoform), which is frequently mutated in WT patients and associated with elevated plasma renin levels and hypertension (26). Mutations or dysregulation of WT1 were further linked to elevated REN expression, hypertension, and exacerbated tumor progression. These findings underscore the synergistic role of WT1 and REN in reinforcing CSC-driven aggressiveness in WT (27, 28).

Spatial and single-cell analyses provided critical insights into REN’s immunosuppressive role. REN expression was mapped to specific tumor cells as well as distinct tumor microenvironmental niches, establishing its involvement in driving NK cell exhaustion. Single-cell communication analyses identified unique signaling axes, including PTN–NCL and COL4A1–CD44, which were exclusive to high-REN-expressing tumor cells and their NK cell interactions. These pathways were absent in tumors with low REN expression, indicating direct engagement between REN-expressing tumor cells and NK cells at both molecular and spatial levels. Spatial transcriptomics confirmed intra-spot colocalization of REN-expressing tumor cells and NK cells, a phenomenon not observed with T cells. These findings suggest that REN promotes immune evasion primarily by suppressing NK cell cytotoxicity rather than affecting T cell functions. Mechanistically, interactions like COL4A1–CD44 activate immune regulatory pathways (such as PI3K/AKT and MAPK signaling) (29), further impairing NK cell-mediated killing. Similarly, PTN–NCL, a signaling axis involved in stem-cell properties and angiogenesis (30), collaborates with immune checkpoints to drive profound immune suppression (31). Additionally, tumor-derived factors such as PGE2 and IL-6 exacerbate NK cell dysfunction, suppressing IFN-γ secretion and inducing an exhausted NK cell phenotype (14, 16). Together, these findings establish REN as a central mediator of tumor stemness and immune evasion and provide a strong rationale for targeting REN to enhance NK cell activity and improve therapeutic outcomes in WT.

The renin gene (REN), encoding the enzyme renin, serves as a central component of the renin-angiotensin system (RAS), which plays critical roles in blood pressure regulation, electrolyte balance, and cellular proliferation. Dysregulation of REN expression has been implicated in diverse pathologies, particularly hypertension, renal diseases, and kidney tumors (32). Renin is a conserved aspartyl protease whose primary function is to cleave angiotensinogen into angiotensin I (Ang I), thereby activating the RAS pathway. Its transcriptional regulation involves intricate, multilayered mechanisms, including the interplay of promoters, enhancers, intronic silencers, and various signaling pathways, such as cAMP, calcium, inflammatory mediators, and nuclear receptors (32). These mechanisms ensure precise expression of REN under both physiological and pathological conditions, maintaining RAS homeostasis (32, 33).

Emerging evidence suggests that beyond its canonical roles in blood pressure control, REN activation contributes to tumor initiation, progression, and the modulation of the tumor immune microenvironment, particularly in renal malignancies (34, 35). In renal cell carcinoma (RCC), REN-mediated activation of the RAS—especially the Ang II/AT1R axis—within the tumor microenvironment has been shown to drive pro-tumorigenic processes such as angiogenesis, inflammation, and metastasis, as well as immune suppression (36). Clinical reports of tumors with aberrant REN expression, including those with secondary hypertension arising from renin-secreting tumors, further support the association between REN and cancer biology (37, 38). Notably, RAS inhibitors (RASi) have demonstrated both antihypertensive and anti-tumor effects, with combinations of RASi and VEGF-targeting agents significantly improving overall survival in metastatic RCC (39).

Mechanistically, REN exerts its oncogenic influence via RAS activation, with the Ang II/AT1R axis promoting proliferation, angiogenesis, fibrosis, metastasis, and immune suppression within the TME. RAS inhibitors mitigate these effects by blocking AT1R signaling, thereby reducing fibrosis, restoring vascular integrity, reprogramming immune cells, and enhancing responsiveness to immunotherapies (34, 35). Preclinical studies combining RASi with chemotherapy or immunotherapy have demonstrated promising therapeutic outcomes, though robust optimization through precision medicine approaches remains essential. As REN represents an upstream regulator of the RAS pathway, targeting REN itself may offer a more precise and effective therapeutic approach, mitigating the tumor-promoting and immunosuppressive effects of RAS activation. Building on our findings, direct modulation of REN activity to reinvigorate NK cell cytotoxicity, as well as targeted inhibition of its downstream signaling pathways, holds the potential to disrupt the immunosuppressive tumor microenvironment and overcome tumor stemness-driven resistance. Such strategies could serve as promising adjuncts to existing therapeutic modalities, paving the way for precision-based interventions aimed at improving clinical outcomes and survival for WT patients.

## Data Availability

The original contributions presented in the study are included in the article/**Supplementary Material**, further inquiries can be directed to the corresponding authors.
