# Supplementary figures and images for: Integrated transcriptomics and machine learning reveal REN as a dual regulator of tumor stemness and NK cell evasion in Wilms tumor progression
Source: Front Immunol. 2025 Jun 4;16:1612987. doi: 10.3389/fimmu.2025.1612987 (PMC12174124; doi:10.3389/fimmu.2025.1612987)

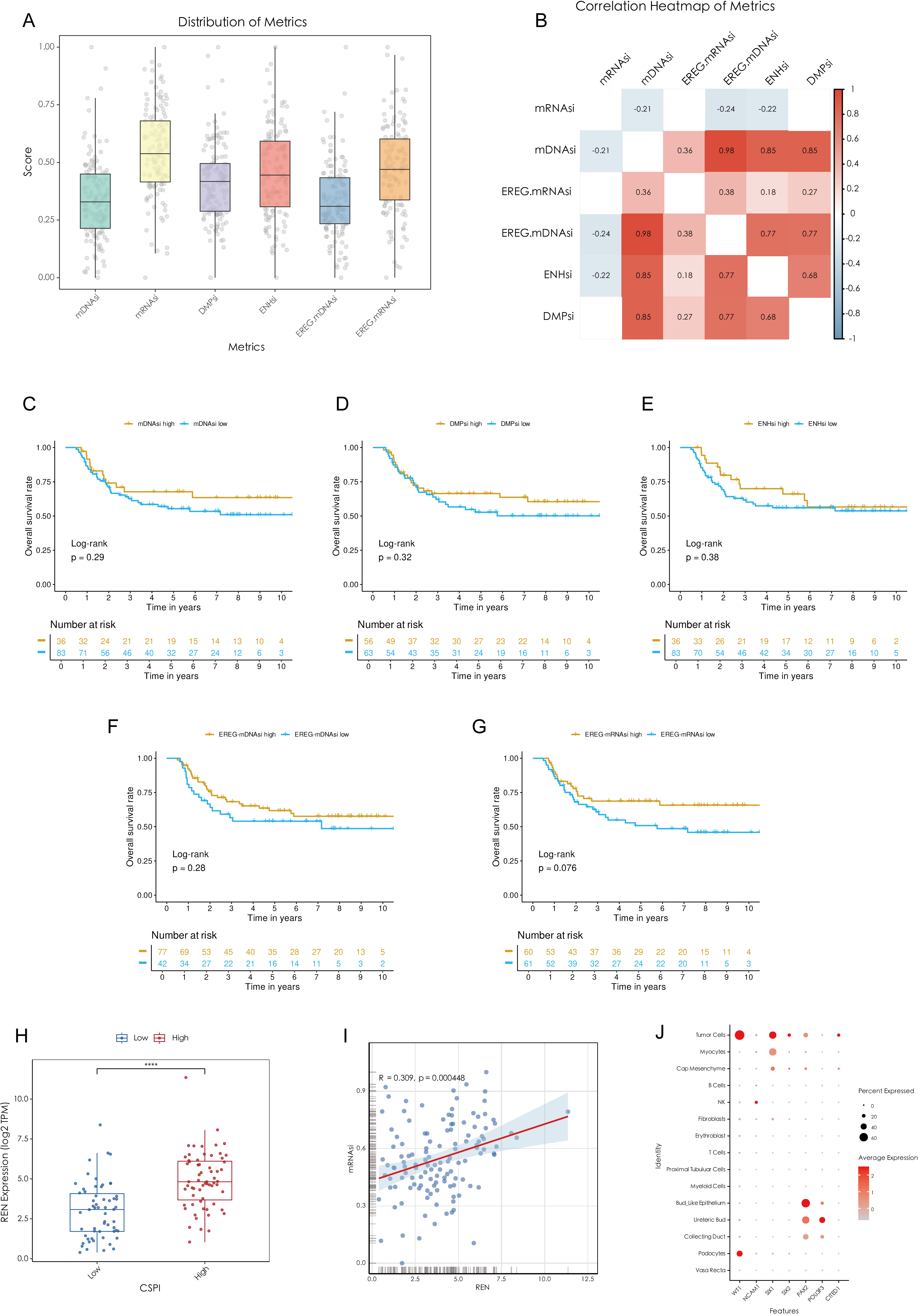

Supplement: Supplementary file 1 [file Image1.tif]
