# Supplementary material for: Integrated transcriptomics and machine learning reveal REN as a dual regulator of tumor stemness and NK cell evasion in Wilms tumor progression
Source: Front Immunol. 2025 Jun 4;16:1612987. doi: 10.3389/fimmu.2025.1612987 (PMC12174124; doi:10.3389/fimmu.2025.1612987)
Supplement: Supplementary file 3 [file DataSheet1.docx]

Integrated Transcriptomics and Machine Learning Reveal REN as a Dual Regulator of Tumor Stemness and NK Cell Evasion in Wilms Tumor Progression

Qingfei Cao^1†^, Junyi Li^1*†^, Changwen Xu^1^, Yunfei Zou^2^, Huihui Tang^1^, Meixue Chen^3*^

^1^ Department of Urology, The First Affiliated Hospital of Jinzhou Medical University, Jinzhou, Liaoning, China

^2^ Department of Cardiology, The First Affiliated Hospital of Jinzhou Medical University, Jinzhou, Liaoning, China

^3^ Department of Pediatric, The First Affiliated Hospital of Jinzhou Medical University, Jinzhou, Liaoning, China

**†These authors contributed equally to this work and share first authorship**

*** Correspondence:**Junyi Li^1^
Email address: [lijunyi.biomed@gmail.com](mailto:leeyee559@163.com)

Meixue Chen^3^

Email address: [chenmeixue@jzmu.edu.cn](mailto:chenmeixue@jzmu.edu.cn)

Supplementary Material

# Supplementary Figures and Tables

## Supplementary Figure

**Supplementary Figure 1. Comparative Analysis of Stemness Metrics and the Role of REN in Wilms Tumor Biology.** (A) Violin plots depicting the distribution of various established stemness metrics: mDNAsi, mRNAsi, DMPsi, ENHsi, EREG.mDNAsi, and EREG.mRNAsi. The metrics were calculated and compared to evaluate tumor stemness within Wilms tumor samples. (B) Correlation heatmap illustrating pairwise relationships between stemness metrics. (C–G) Kaplan-Meier survival curves analyzing the prognostic significance of tumor stemness metrics, including (C) mDNAsi, (D) DMPsi, (E) ENHsi, (F) EREG mDNAsi, and (G) EREG mRNAsi. Log-rank tests were performed to assess the prognostic utility of each metric in stratifying Wilms tumor patient outcomes. (H) REN expression across Cancer Stemness Prognostic Index (CSPI)-defined patient risk groups. (I) Scatter plot showing the positive correlation between REN expression and mRNAsi across Wilms tumor samples. (J) Dot plot showing the expression levels and percent expression of canonical tumor cell marker genes across different cell types within the Wilms tumor microenvironment.

## Supplementary Table

**Supplementary Table 1.** Primer and siRNA Sequences used in this study.
